# Hypergravity Load Modulates Acetaminophen Nephrotoxicity via Endoplasmic Reticulum Stress in Association with Hepatic microRNA-122 Expression

**DOI:** 10.3390/ijms22094901

**Published:** 2021-05-05

**Authors:** Hong-Min Wu, Sang-Gil Lee, Choong-Sik Oh, Sang-Geon Kim

**Affiliations:** 1 College of Pharmacy, Seoul National University, Seoul 08826, Korea; minmin0210@snu.ac.kr (H.-M.W.); elen7020@snu.ac.kr (S.-G.L.); 2 Aerospace Medical Center, ROKAF, Cheong-ju 360-842, Korea; ocs2276@naver.com; 3 College of Pharmacy and Integrated Research Institute for Drug Development, Dongguk University-Seoul, Goyang-si, Gyeonggi-Do 10326, Korea

**Keywords:** hypergravity, ER stress, kidney injury, miR-122, adaptation

## Abstract

Hypergravity conditions may subject the kidney to intrinsic stress and lead to hemodynamic kidney dysfunction. However, the mechanisms underlying this phenomenon remain unclear. Accumulation of unfolded proteins in the endoplasmic reticulum (i.e., ER stress) is often observed in kidney diseases. Therefore, this study investigated whether hypergravity stress alters acetaminophen-induced renal toxicity in vivo, as well as the molecular mechanisms involved in this process. C57BL/6 mice were submitted to one or three loads of +9 Gx hypergravity for 1 h with or without acetaminophen (APAP) treatment. The protein levels of cell survival markers, including pAKT and pCREB, were decreased in the kidney after acetaminophen treatment with a single hypergravity load. Additionally, the combined treatment increased kidney injury markers, serum creatinine, and *Bax*, *Bcl2*, and *Kim-1* transcript levels and enhanced ER stress-related markers were further. Moreover, multiple hypergravity loads enabled mice to overcome kidney injury, as indicated by decreases in serum creatinine content and ER stress marker levels, along with increased cell viability indices. Similarly, multiple hypergravity loads plus APAP elevated miR-122 levels in the kidney, which likely originated from the liver, as the levels of primary miR-122 increased only in the liver and not the kidney. Importantly, this phenomenon may contribute to overcoming hypergravity-induced kidney injury. Taken together, our results demonstrate that APAP-exposed mice submitted to a single load of hypergravity exhibited more pronounced kidney dysfunction due to increased ER stress, which may be overcome by repetitive hypergravity loads presumably due to increased production of miR-122 in the liver. Thus, our study provides novel insights into the mechanisms by which hypergravity stress plus APAP medication induce kidney injury, which may be overcome by repeated hypergravity exposure.

## 1. Introduction

Since the beginning of manned space missions in 1961, technological advancement has reached a point where space travel has become within reach of civilians and trained astronauts alike (e.g., Space-X project) [1,2]. However, despite recent technological advances and prospects, many factors, such as gravitational changes, ionizing radiation, and physiological/psychological stressors, threaten the health of astronauts outside of the Earth’s atmosphere [3]. Particularly, the effects of gravitational changes associated with space flight on living organisms remain poorly documented due to a lack of ground-based facilities and experimental limitations.

Exposure to spaceflight conditions could result in physical and physiological stress due to gravitational alterations [1,2,3,4,5,6,7,8,9,10]. The greatest gravitational change that living organisms experience during escape and re-entry into the Earth’s atmosphere is the high gravity applied during the ascent of the launch vehicle and the rapid change to weightlessness experienced thereafter. Jetfighter pilots also experience this phenomenon with impacts of up to +9 G or more [6]. The gravity-associated stress experienced by living organisms varies depending on the research model, object, and observation index. Therefore, a systematic approach is required to facilitate data interpretation and ensure compatibility.

Increased secretory load or the accumulation of misfolded proteins in the endoplasmic reticulum (ER) causes ER stress in the cell due to an imbalance between ER capacity and protein-folding load [11,12]. Increased intrarenal fatty acid levels may reportedly trigger oxidative and ER stress, which in turn activates the unfolded protein response (UPR), which ultimately leads to cell apoptosis if left unresolved. The ER stress response is mediated by sensors of three canonical pathways located in the ER membrane: protein kinase RNA (PKR)-like ER kinase (PERK), inositol-requiring protein-1α (IRE1α), and activating transcription factor 6 (ATF6) [13,14,15]. Multiple clinical and experimental studies have demonstrated that a maladaptive ER response is mechanistically linked to the pathogenesis of various diseases accompanying cell death [16,17,18,19,20,21,22].

Due to the external changes and harmful stimuli associated with spaceflight, additional pharmaco/toxicological research is critical for developing strategies to allow spacecraft pilots and passengers to successfully adapt to these stimuli, avoid damage, and maintain homeostasis for survival. Acetaminophen (APAP) is a commonly prescribed analgesic and antipyretic agent [23]. APAP has a broad safety margin at therapeutic doses [24]. However, this compound may cause nephrotoxicity and hepatotoxicity under certain physiological conditions, particularly in overdose cases. Blood urea nitrogen (BUN) and serum creatinine are used as indicators of kidney dysfunction [25,26,27]. In one of our previous studies, we found that +9 G hypergravity stress increases BUN and serum creatinine contents, suggesting induction of kidney dysfunction due to gravitational stress [6].

Given the lack of research on the joint effect of hypergravity and medications on organ functions under outer space environment conditions, this study investigated whether single and multiple loads of hypergravity stress affect APAP nephrotoxicity and hepatotoxicity. Our findings demonstrated that kidney function was affected when APAP was coupled with hypergravity stimulation, resulting from augmented ER stress in the cell. Moreover, we explored whether multiple hypergravity loads could ameliorate APAP toxicity via adaptation, after which we examined the molecular basis for APAP toxicity and adaptation, focusing on miR-122 and primary miR-122 (pri-miR-122) levels in the kidney and liver. Therefore, our findings may provide important insights into the biological basis of hypergravity training and medication.

## 2. Results

### 2.1. Kidney Injury Associated with a Single Hypergravity Load Coupled with APAP Treatment

**Figure 1:**
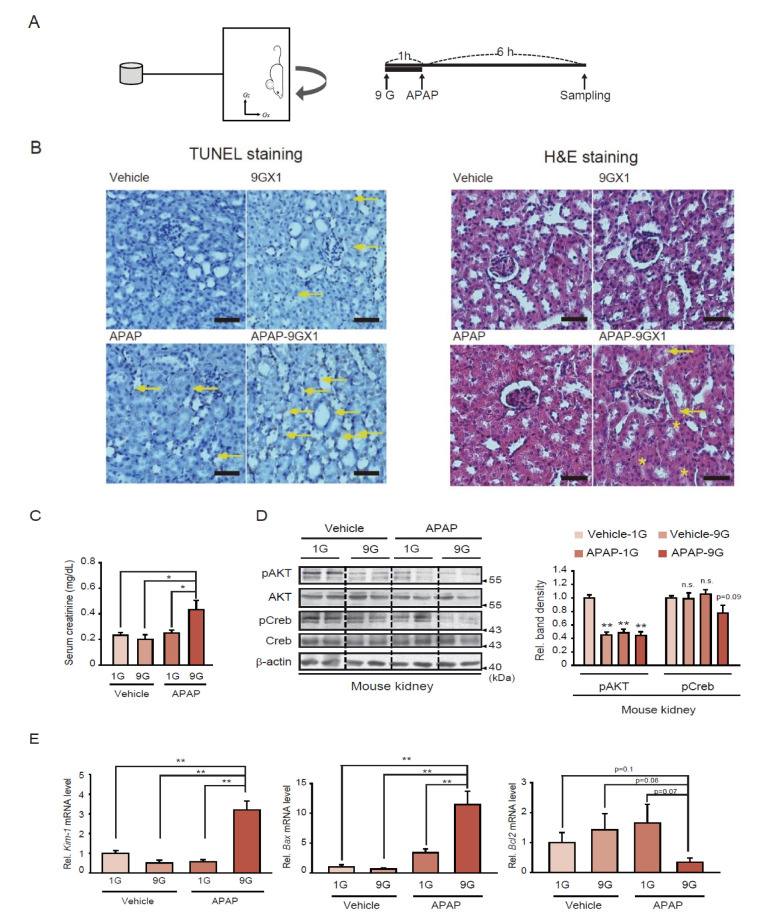
Increased kidney dysfunction in mice exposed to a single hypergravity load coupled with APAP treatment. (**A**) Schematic of hypergravity and APAP treatments. (**B**) TUNEL and H&E staining of kidney sections. Male C57BL/6 mice were exposed to a single +9 Gx load for 1 h, followed by APAP treatment (500 mg/kg BW, i.p.), and sacrificed 6 h afterward. TUNEL and H&E staining was performed for two samples per treatment condition (randomly selected from 6 samples). Scale bar: 100 μm. The arrows indicate TUNEL-positive cells. The asterisks in the H&E staining images indicate desquamation of the tubular epithelium, and arrows indicate nuclear swelling and vacuolization of the tubular epithelium in necrotizing tubular epithelia. (**C**) Serum creatinine contents in the same mice as in panel B (*n* = 6/group). (**D**) Immunoblots for pAKT and pCREB in renal medulla homogenates from the same mice as in panel B (representative blots are presented). Quantifications were conducted for six samples per group. (**E**) qRT-PCR assays for *Kim-1*, *Bax*, and *Bcl2* in the renal cortex homogenates. The data in C-E represent the mean ± SEM (statistical differences were determined via the one-way ANOVA; * *p* < 0.05, ** *p* < 0.01, n.s., not significant).

A hypergravity animal model was employed to understand the effect of a single load of +9 Gx stress alone or in combination with APAP treatment, after which morphological and molecular analyses of the kidney tissue were conducted (Figure 1A). Terminal deoxynucleotidyl transferase dUTP nick end labeling (TUNEL) staining and hematoxylin and eosin (H&E) staining analyses revealed an increased intensity of TUNEL-positive cells and disorganized tubular epithelium cells in mice exposed to a single hypergravity load coupled with APAP treatment, compared to hypergravity or APAP treatment alone (Figure 1B). Similarly, serum creatinine (i.e., a serum marker for kidney injury) content was significantly increased in the animals exposed to the combined treatment, whereas hypergravity or APAP treatment alone rendered no significant effects (Figure 1C).

pAKT and pCREB levels were then analyzed as indicators of cell survival. As expected, pAKT (Ser473) levels were suppressed by either a single load of hypergravity stress, APAP treatment alone, or their combination (Figure 1D). Moreover, a single hypergravity load coupled with APAP treatment seemed to lower pCREB levels to a greater extent than each treatment alone. Similarly, quantitative reverse transcription-polymerase chain reaction (qRT-PCR) assays provided strong evidence that a single hypergravity load coupled with APAP markedly enhanced the levels of *Kim-1* (i.e., a kidney injury marker) and *Bax* (i.e., a proapoptotic gene) transcripts compared to either a single load of hypergravity or APAP treatment alone (Figure 1E, left and middle). Moreover, the transcript level of *Bcl2* (i.e., an anti-apoptotic gene) was significantly reduced in animals exposed to both hypergravity and APAP (Figure 1E, right). Based on these results, we hypothesized that a single hypergravity load coupled with APAP treatment might synergistically exacerbate kidney injury and dysfunction.

### 2.2. Induction of ER Stress by a Single Hypergravity Load Coupled with APAP Treatment

**Figure 2:**
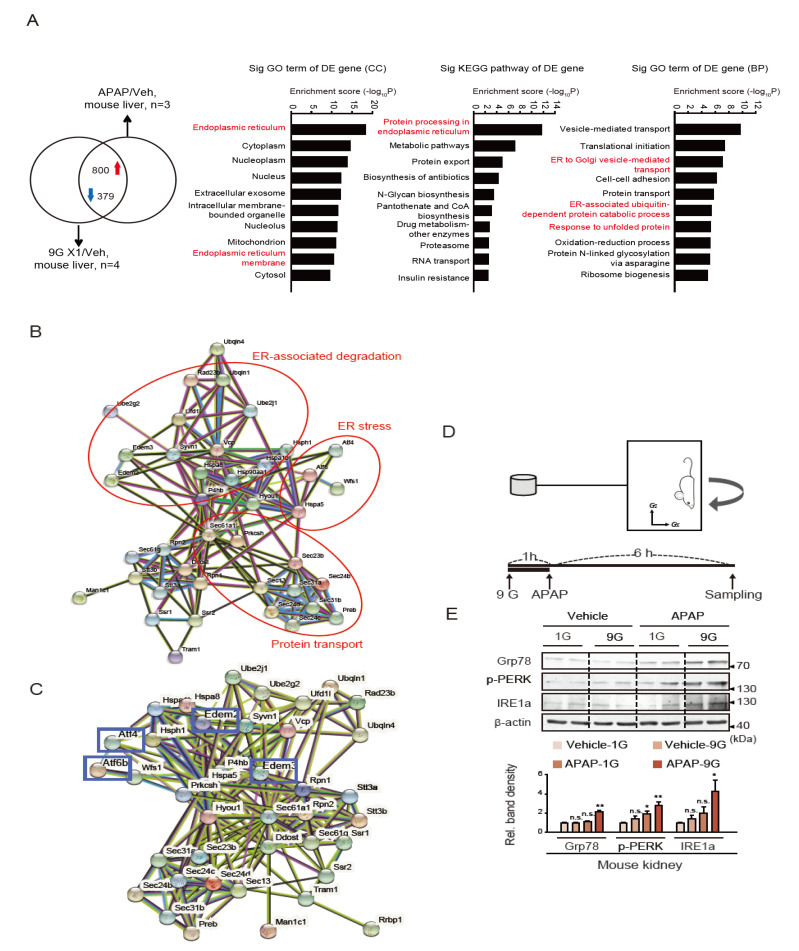
Increased ER stress in the kidneys of mice exposed to a single hypergravity load coupled with APAP treatment. (**A**) Bioinformatic analysis of genes affected by +9 Gx load and APAP in mouse liver. The Venn diagram illustrates the DEG overlap according to the cDNA microarray data obtained from the analysis of mouse livers [6] and RNA-seq analysis data for the liver of mice treated with APAP [30]. DEG pathway enrichment analysis was conducted using the DAVID database. (**B**) Protein-protein interaction network. An interaction network for the DEGs of ER-related enriched pathways (marked in red in panel A) was constructed using the STRING database. (**C**) The network of ER stress-related gene clusters. Boxes indicate well-recognized ER stress-associated genes. (**D**) Schematic of hypergravity and APAP treatments. (**E**) Immunoblots for representative ER stress marker proteins. Assays were performed on renal medulla homogenates from the same mice as in Figure 1B (representative immunoblots are presented). Quantifications were done for six samples per group. The data represent the mean ± SEM (statistical difference was determined via the one-way ANOVA; * *p* < 0.05, ** *p* < 0.01, n.s., not significant).

Next, we used the dataset obtained from the liver of mice exposed to a single hypergravity load and APAP treatment to identify the Gene Ontology (GO) terms significantly affected by this combination (Figure 2A). The analysis of the GO terms using the DAVID database enabled identifying DEG enrichment in ER-associated pathways. Additionally, alterations in ER stress-associated markers, such as Atf4, Atf6, Wfs1, and Hspa5, were identified via STRING database network analysis (Figure 2B). Particularly, the gene clusters associated with ER-associated degradation, ER stress, and protein transport were functionally linked to each other. The network of ER stress-related 40 genes is additionally shown (Figure 2C); Of these, ER degradation enhancer, mannosidase alpha-like 2,3 (Edem2,3), Atf4 and Atf6 are well-recognized ER stress target genes.

According to the immunoblotting assays, a combined exposure to a single hypergravity load and APAP significantly enhanced the levels of ER stress markers in mice, including Grp78, p-PERK, and IRE1a, whereas hypergravity or APAP treatment alone only increased their expression modestly (Figure 2D,E). Therefore, a single load of hypergravity stress coupled with APAP treatment may synergistically exacerbate ER stress in the kidney, resulting in kidney dysfunction.

Next, we examined whether a single hypergravity load coupled with APAP treatment altered cell viability and ER stress markers in other organs, such as the liver and skeletal muscle, for comparison (Supplementary Figure S1B). In the liver, pAKT and pCREB levels were decreased by either +9 Gx load or APAP treatment and were further decreased by their combination (Supplementary Figure S1B, left). In contrast, the combined treatment (Supplementary Figure S1B, right) increased ER stress marker levels. In skeletal muscle, either hypergravity or APAP treatment alone similarly decreased pAKT, but the combined treatment did not further enhance the effect (Supplementary Figure S1C, left). The level of pCREB was affected by the combined treatment. Moreover, ER stress was exacerbated by the combined treatment in skeletal muscle (Supplementary Figure S1C, right). These results suggest that hypergravity load coupled with APAP treatment may systemically decrease the expression of cell survival biomarkers with reciprocal increases in ER stress marker levels in the liver and skeletal muscle, and the kidney. 

### 2.3. Repeated +9 Gx Hypergravity Exposure Attenuates APAP-Induced Renal Injury via Preconditioning

**Figure 3:**
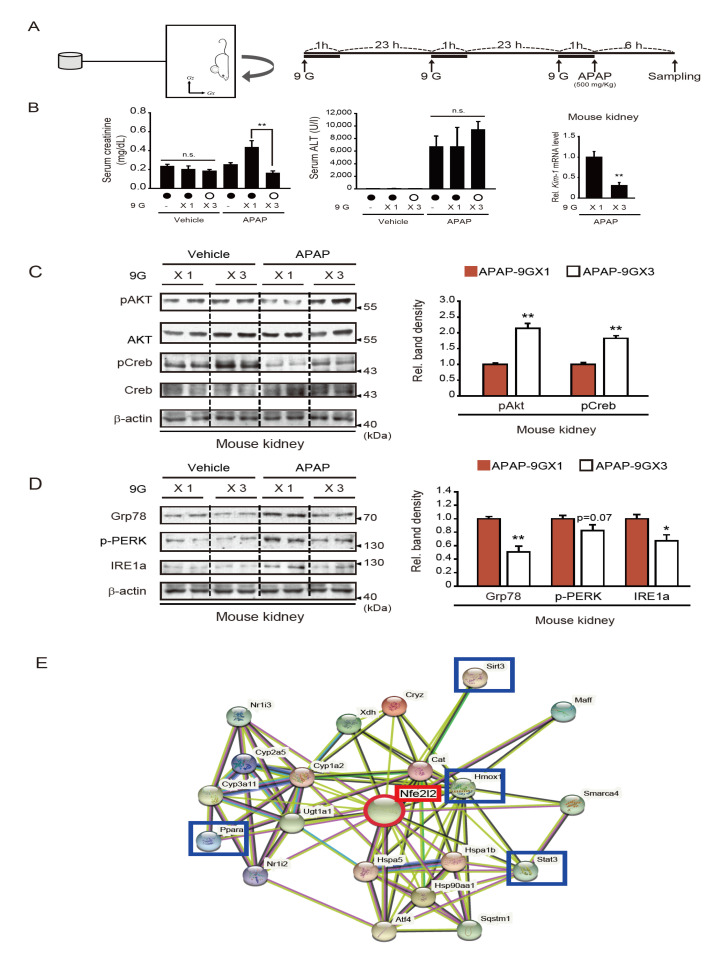
Effects of hypergravity preconditioning on kidney dysfunction, cell survival marker expression, and ER stress induced by APAP treatment. (**A**) Schematic of hypergravity and APAP treatments. (**B**) Serum creatinine contents, ALT activities, and qRT-PCR assay results for Kim-1 in the renal cortex. Male C57BL/6 mice (*n* = 5 for APAP-9 GX3 group, and *n* = 6 for the other groups) were subjected to a single +9 Gx load for 1 h (9 GX1) or multiple +9 Gx loads for 1 h on three consecutive days (9 GX3), followed by a single administration of APAP (500 mg/kg BW, i.p.); the mice were sacrificed 6 h afterward. (**C**,**D**) Immunoblots for pAKT and pCREB (**C**) and ER stress marker proteins (**D**). Assays were conducted on the same renal medulla homogenates as above (*n* = 5 for the APAP-9 GX3 group; *n* = 6 for the other groups). (**E**) The network of genes linked to Nrf2 in the liver of mice treated with APAP and hypergravity. Blue boxes indicate representative cell-survival marker proteins. The data were reported as the mean ± SEM (statistical differences were determined via the one-way ANOVA; * *p* < 0.05, ** *p* < 0.01, n.s., not significant).

We previously reported that multiple hypergravity loads (i.e., three loads) induce an adaptive response to hypergravity-induced stress, resulting in no hepatic injury in mice [6]. Here, we examined the effect of multiple hypergravity loads coupled with APAP treatment on kidney and liver functions and compared the outcomes with those of a single hypergravity load coupled with APAP (Figure 3A). As mentioned above, the latter combination significantly increased serum creatinine content (Figure 3B, left). In contrast, multiple hypergravity loads coupled with APAP did not induce renal dysfunction, as determined by serum creatinine analysis, but rather slightly improved kidney function compared to APAP treatment alone. This suggests that multiple hypergravity loads may exert an adaptive cytoprotective effect in the kidney against APAP. Mice treated with either APAP alone or in combination with a single hypergravity load also exhibited a marked increase in serum alanine aminotransferase (ALT) activity (Figure 3B, middle). This was also supported by a decrease in the pAKT level and increases in ER stress markers in the liver (Supplementary Figure S1B). In contrast with the cytoprotective effects observed in the kidney, the increases in ALT activity (i.e., an indicator of liver injury) were not abrogated in the group exposed to both hypergravity and APAP (Figure 3B, middle) compared to the hypergravity-trained group. Skeletal muscle tissues also exhibited some changes in the level of pAKT and ER stress markers (Supplementary Figure S1C). The qRT-PCR assays for *Kim-1* in the kidney highlighted the renal protective effects of hypergravity load preconditioning. Specifically, *Kim-1* transcript levels were lower in the preconditioned group than the group exposed to a single hypergravity load coupled with APAP (Figure 3B, right).

To further investigate the beneficial effects of multiple hypergravity loads on kidney function, cell survival rates and ER stress markers were analyzed in kidney samples. As expected, the levels of pAKT and pCREB (i.e., survival markers) were both lower in mice exposed to a single hypergravity load coupled with APAP compared to either condition alone; however, this effect was not seen in mice exposed to multiple hypergravity loads coupled with APAP (Figure 3C, left and right). Similarly, the expression levels of ER stress markers, including Grp78, p-PERK, and IRE1a, were all greater in the mice exposed to a single hypergravity load coupled with APAP compared to the individual exposures (Figure 3D, left). Again, these changes were not observed in animals subjected to multiple hypergravity loads. Consequently, the latter group exhibited decreases in ER stress marker protein levels than the former group (Figure 3D, right). In our previous study, Nrf2 plays a key role in inhibiting ROS production elicited by hypergravity stress in the model of multiple hypergravity loads [6]. Given the role of Nrf2 and the established association of ER stress and ROS generation, we further analyzed the dataset obtained from the liver of mice treated with APAP using the STRING program and found that Nrf2 transcript level is closely linked to cell-survival marker proteins, including Sirt3, Ppara, Stat3, and Hmox1. The network obtained from the additional bioinformatic analysis was also shown (Figure 3E). Together, our results provide evidence that multiple loads of hypergravity protect the kidney from APAP-induced kidney injury in association with decreased ER stress.

### 2.4. Adaptive Increases in miR-122 Levels in the Kidney after Preconditioning with Multiple Hypergravity Loads

**Figure 4:**
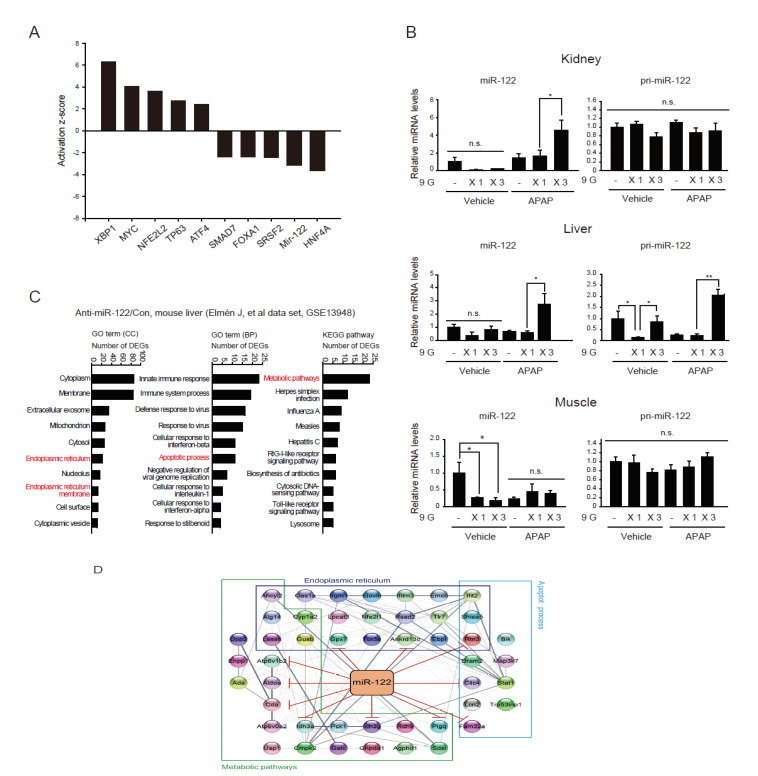
miR-122 and primary miR-122 levels in major organs of mice exposed to combined treatments of hypergravity load(s) and APAP and putative target genes of miR-122. (**A**) The upstream regulator analysis using Ingenuity Pathway Analysis (IPA) software (QIAGEN) from the 1179 overlapping DEGs (Figure 2A) that commonly up- (800 genes) or down- (379 genes) regulated by a single hypergravity load and by an APAP treatment. (**B**) qRT-PCR assays for mature miR-122 (miR-122) and primary miR-122 (pri-miR-122) in the renal cortex, liver, and tibialis anterior muscle of the same mice as in Figure 3B (*n* = 5 for the APAP-9 GX3 group; *n* = 6 for the other groups). (**C**) Bioinformatic analysis of genes affected by miR-122 inhibition. A public GEO database (GSE13948) obtained using mouse livers subjected to chemically modified antisense oligonucleotides for the inhibition of miR-122 was analyzed. DEG pathway enrichment was analyzed using the DAVID database. (**D**) Protein-protein interaction network for the DEGs associated with ER-related enriched pathways (i.e., endoplasmic reticulum, endoplasmic reticulum membrane, apoptosis, and metabolism) using the STRING database. The solid red lines indicate genes containing 3′-UTR-binding sequences for miR-122. The data in B represent the mean ± SEM (statistical difference was determined via the one-way ANOVA; * *p* < 0.05, ** *p* < 0.01, n.s., not significant).

Next, we sought to identify the mechanisms underlying the selective cytoprotective effect of multiple hypergravity against APAP toxicity to the kidney by studying the molecules associated with hypergravity exposure and APAP adaptation. Since the upstream regulators control multiple downstream target genes, we first predicted representative transcription factors using Ingenuity pathway analysis (IPA) software (QIAGEN). Of the 1179 DEGs shown in Figure 2A (800 and 379 up- and downregulated genes, respectively), the predicted most affected genes were listed (Figure 4A): ER stress regulators (XBP1 and ATF4) and pro-apoptosis related regulator (TP63) might be upregulated, while antiapoptosis-related regulators (HNF4A, SRSF2, FOXA1, and SMAD7) being downregulated. This outcome supports the effect of ER stress on kidney cell dysfunction.

In the above database analysis, miR-122 was additionally predicted as one of the highly inhibited upstream regulators. Given the above-described tissue-specific protective effect of multiple hypergravity loads and our previous findings on microRNA downregulation (e.g., miR-122) during acute kidney injury, coupled with the increased abundance of miR-122 in the liver and its supply to other organs [6,28,29], we sought to measure miR-122 levels in the kidney and liver of animals subjected to single or multiple hypergravity loads and APAP treatment.

MiR-122 levels in the kidney were minimally affected by either a single or multiple loads of hypergravity or APAP treatment. Interestingly, however, multiple hypergravity loads coupled with APAP treatment significantly increased miR-122 content in the kidney than individual treatments (Figure 4B, upper). In contrast, other microRNAs previously identified in kidney tissue, such as miR-10b*, miR-30e, miR-193, and miR-26a, only showed minimal changes (data not shown). The primary miR-122 transcript (pri-mir-122) levels in the kidney were not significantly different among the treatment groups (Figure 4B, upper), which suggested the transport of pri-mir-122 miRNA to the kidney from other organ(s).

Given that miR-122 expression was most abundant in the liver and was not affected in the kidney, we examined hepatic miR-122 and pri-miR-122 levels in the liver. We found that multiple hypergravity loads coupled with APAP treatment significantly increased miR-122 expression in the liver, whereas either a single or multiple hypergravity loads or APAP treatment alone had no significant effects on miR-122 expression. This pattern change was very similar to that in the kidney (Figure 4B, middle). Moreover, either a single load of hypergravity or APAP treatment alone lowered the basal pri-miR-122 level in the liver, and this was not seen in animals subjected to multiple loads of hypergravity (Figure 4B, middle). More importantly, coupling multiple loads of hypergravity with APAP treatment significantly enhanced the level of hepatic pri-miR-122 compared to the controls or the respective individual treatments.

As a control, we further examined miR-122 and pri-miR-122 levels in skeletal muscle and found that miR-122 levels were diminished by either a single or multiple loads of hypergravity (Figure 4B, lower). The animals subjected to APAP with either a single or multiple loads of hypergravity combined with APAP exhibited no significant changes among the different groups. Pri-miR-122 levels remained unaltered in skeletal muscle. Other miRNAs, such as miR-1192, 3473c, and 1933, were slightly suppressed by a single hypergravity exposure event or were minimally affected by multiple loads in the kidney or skeletal muscle (Supplementary Figure S2A,B).

Finally, given the importance of miR-122 in the liver and its role in systemic homeostasis, we analyzed differentially expressed genes (DEGs) from a GEO database (GSE13948) available in the public domain, which was obtained using chemically modified antisense oligonucleotides to specifically inhibit endogenous miR-122 in the liver. Using this dataset, we performed pathway enrichment analysis using the DAVID database. GO analysis indicated that the DEGs were enriched in pathways related to ER, ER membrane, apoptosis, and metabolism (Figure 4C). Moreover, network analysis using the STRING database elucidated 11 genes among the 47 genes in the network that were potential miR-122 targets, given that their 3′-UTR regions exhibited predicted binding sequences for miR-122 (Figure 4D).

Together, our results suggest that multiple hypergravity loads and APAP treatment promote pri-miR-122 production in the liver, which may then be transported to the kidney tissue via blood circulation in a context-dependent manner for regulating ER-associated physiological functions.

## 3. Discussion

The assessment of gravitational stress would provide a basis for quantifying biological stress in response to hypergravity and gravity changes, which may facilitate developing preventive strategies and treatments. We previously demonstrated that a single hypergravity load (+9 Gz) might cause compressional injury stress on the liver [6]. Given that the kidney is another pressure-sensitive organ that filters out waste substances in the blood and some metabolites in the blood can be a risk factor for kidney damage when taking drugs, we examined whether the addition of APAP intake stress alters kidney damage in organisms exposed to hypergravity stress. Our findings demonstrate that combining +9 Gx hypergravity and APAP enhances kidney dysfunction, as shown by the outcomes of the staining intensity analysis of TUNEL-positive cells, and alters the levels of *Bax* and *Bcl2* transcripts, as well as serum creatinine and *Kim-1* mRNA. In line with these observations, disorganization of tubular epithelia was also observed, thus indicating renal cell damage. These findings were supported by the observed alterations in the levels of cell viability and apoptosis markers.

Importantly, our study also determined that multiple hypergravity loads protected the kidney from APAP-induced injury via preconditioning. This idea was supported by the changes in renal cell viability biomarkers and *in vivo* renal injury markers (i.e., creatinine and *Kim-1*) observed herein. However, intriguingly, this preconditioning effect did not effectively protect the liver from APAP, suggesting that preconditioning only provides kidney-selective cytoprotection. Previous studies have proposed that multiple hypergravity loads allow the liver to protect itself from compressional injury and hypergravity stress [6]. However, our findings demonstrate that hypergravity preconditioning coupled with APAP treatment failed to elicit an adaptive response in the liver, unlike in the kidney.

Moreover, our results demonstrated that the combined treatment with hypergravity and APAP increases ER stress, supported by both GO biological process analyses and experimental approaches. Again, the enhanced ER stress in the kidney was also alleviated when the mice were previously exposed to repetitive hypergravity stress. When APAP was administered with a single gravity load, the PERK-mediated pathway was strongly upregulated in the kidney. Activation of XBP1s under IRE1a modulates ER folding capacity and mitigates the detrimental effects of unfolded proteins, whereas sustained production of XBP1 induces cell death and organ dysfunction [30,31,32,33]. Our findings supported the notion that IRE1a was further activated by the combined treatment [21,22,34]. However, there was no significant change in ATF6 protein levels. Therefore, it appears that ER stress signals are enhanced through the PERK and IRE1a pathways in the kidney when hypergravity stress is applied with APAP treatment. In the ER stress response, we also found kidney-selective adaptation after multiple loads of hypergravity against APAP. The lack of changes in ER stress relief and cell viability markers in the liver paralleled each other. Thus, it is highly likely that the functional changes observed in the kidney and liver after hypergravity load(s) coupled with APAP treatment were mediated by ER stress.

MiRNAs may be responsible for regulating up to 30% of the human protein-coding genes [35]; however, other potential targets may also exist and be involved in regulating the adaptation to hypergravity exposure and APAP intoxication. We then turned our focus to miR-122 to identify the mechanisms that mediate tissue-selective adaptation against APAP intake, as miR-122 is the most abundant miRNA in the liver and may play a role in systemic homeostasis. Interestingly, miR-122 and pri-miR-122 levels increased in the liver when combined with repeated hypergravity stress and APAP treatment, consistent with our observations in the kidney. Therefore, it is highly likely that multiple loads of hypergravity in conjunction with APAP upregulate pri-miR-122 levels in the liver, and the circulating mature form of miR-122 generated from pri-miR-122 in the liver may contribute to an adaptive response in the kidney against APAP intoxication, which exhibited no change in pri-miR-122 level. This hypothesis is supported by analyzing a public GEO database (GSE13948) and DEG enrichment in pathways associated with ER biology, apoptosis, and metabolism. Network analysis using the STRING database elucidated 11 genes out of 47 genes in the network that were potential miR-122 targets, as they exhibited potential miR-122-binding sequences in their 3′-UTR region. Therefore, it is highly likely that liver-secreted miR-122 plays a role in regulating homeostasis in the kidney and contributes to the expression of genes protecting renal cells from APAP toxicity.

In this study, miR-122 was also significantly decreased in skeletal muscle after a single hypergravity stress event, and this decrease was maintained after repeated hypergravity stimulation. Furthermore, pri-miR-122 exhibited lower expression levels in skeletal muscle even after three multiple loads. Therefore, it seems that miR-122, which was reduced in skeletal muscle, did not recover after hypergravity stress preconditioning. A combination of APAP and repetitive hypergravity loads induced mild ER stress in skeletal muscle but was not strong enough to result in muscle toxicity. Conversely, the combination of APAP and hypergravity stress appeared to improve the cell viability index, which is consistent with a study that reported that APAP administration increased muscle mass and strength [36,37].

AMPK is a key intracellular energy sensor activated through phosphorylation and is associated with regulating whole-body homeostasis [38,39]. Previously, we found that a single hypergravity load represses liver AMPK activity, and that this can be restored by multiple loads to hypergravity [6]. In the current study, we examined whether there were similar effects on AMPK in the kidney and skeletal muscle of hypergravity-subjected mice. In the kidney, AMPK phosphorylation was decreased by multiple hypergravity loads (Supplementary Figure S3). AMPK was inhibited by a single hypergravity load in skeletal muscle, and the repressed AMPK was recovered by repetitive training loads (data not shown). This outcome was similar to that in the liver but differed from that in the kidney. This suggests that energy metabolism may also be differentially regulated by hypergravity exposure(s) in a context-dependent manner. Several miRNAs, including miR-1192, miR-1933, and miR-3473c, were also found to be sensitive to hypergravity changes in the liver (unpublished data). Only miR-1192 was significantly decreased by a single hypergravity load in both kidney and skeletal muscle and was recovered after multiple loads (Supplementary Figure S2B). It has been shown that miR-1192 inhibits HMGB1 mRNA translation [40], and knockdown of HMGB1 increases AMPK activity [41]. Therefore, miR-1192 may indirectly upregulate AMPK activity.

Therefore, the underlying mechanisms of hypergravity adaptation must be elucidated in aeronautical science and industry to ensure space travel safety. The outcomes of this study show that joint exposure to hypergravity stress and APAP may exacerbate kidney dysfunction through ER stress, which may be abrogated by multiple preconditioning loads (Supplementary Figure S4). Moreover, our data demonstrated the importance of miR-122 in this phenomenon, thus providing new information on the adaptations of the kidney to hypergravity stress and medication during space travel.

## 4. Materials and Methods

### 4.1. Experimental Animals

Animal experiments were conducted under the Institutional Animal Use and Care Committee guidelines at Seoul National University. Eight-week-old male C57BL/6 mice were purchased from Charles River Orient (Seoul, Korea) and housed at 20 ± 2 °C with 12 h light/dark cycles and relative humidity of 50 ± 5% (Tecniplast, Varese, Italy). The cages were supplied with filtered pathogen-free air, and food (Purina, Seongnam, Korea) and water were available ad libitum. All mice were acclimatized for 1 week at the Animal Center for Pharmaceutical Research (Seoul National University).

### 4.2. Centrifugation Experiment

The mice were exposed to short-term hypergravity at +9 Gx for 1 h using a small animal centrifuge at the Aerospace Medicine Research Center (Cheongju, Korea). The mice were placed inside a cylindrical plastic restraint device that allowed +9 Gx to be delivered along the rostrocaudal axis when mounted in the centrifuge. A cage-mounting module was attached at the end of an arm that allowed for one degree of freedom, thereby ensuring that the net gravity field was perpendicular to the floor of the restraint device [6]. The mice were sacrificed 6 h after exposure to hypergravity with or without a single dose of APAP (i.p., 500 mg/kg BW, i.p.) treatment [42]. The dose of APAP was chosen to induce oxidative stress and ER stress. Different sets of animals were submitted to daily hypergravity exposure for three days, after which the tissue samples were obtained 6 h after the last load (Figure 3A). The behavior of the mice throughout the centrifugation experiments was monitored with a charge-coupled device (CCD) camera.

### 4.3. Blood Chemistry

Serum creatinine and ALT activity contents were analyzed using a Spectrum automatic blood chemistry analyzer (Abbott Laboratories, Abbott Park, IL, USA).

### 4.4. Hematoxylin and Eosin Staining

Upon slicing the kidney samples, the tissue slices were fixed in 10% buffered-neutral formalin for 6 h. The samples were then stained with H&E, as previously described [43].

### 4.5. TUNEL Assays

The TUNEL assay was conducted as previously described [44]. Briefly, the assay was performed using the in situ S7100 ApopTag apoptosis detection kit, according to the manufacturer’s instructions.

### 4.6. Immunoblot Analysis

Immunoblot analysis was performed as previously described [45]. Equal sample loads were verified via β-actin immunoblotting. Band intensities were quantified using Adobe Photoshop CS6 (Adobe Systems, San Jose, CA, USA). Antibody information is provided in Supporting Table S1.

### 4.7. Real-Time Polymerase Chain Reaction Assays

Real-time PCR was conducted using a Light Cycler DNA master SYBR green-I kit (Light-Cycler 2.0; Roche, Mannheim, Germany) according to the manufacturer’s instructions. Total RNA was extracted using Trizol reagent (Invitrogen, Carlsbad, CA, USA) and reverse-transcribed to obtain complementary DNAs (cDNAs). qRT-PCR was performed using ABI StepOne Plus real-time PCR system (Thermo Fisher Scientific, Waltham, MA, USA) and SYBR Premix Ex Taq II kit (Takara Bio, Shiga, Japan). The relative mRNA levels were normalized based on actin levels. Mature microRNAs were amplified using each specific primer and the miScript SYBR Green polymerase chain reaction kit (Qiagen, Valencia, CA, USA). Transcripts of U6 small nuclear RNA (U6) were quantified for normalization of microRNA levels. The primers used in qRT-PCR assays are listed in Supporting Table S2.

### 4.8. RNA Quality Check

For quality control, RNA purity and integrity were evaluated based on OD 260/280 ratios and analyzed with an Agilent 2100 Bioanalyzer (Agilent Technologies, Palo Alto, CA, USA).

### 4.9. Integrative Network Analysis

DEGs with statistical significance in the livers of single hypergravity-loaded mice (*p* < 0.1) [6] and APAP-treated mice (*p* < 0.05) [46] were compared, and genes upregulated or downregulated in both database were selected. For another analysis, a public GEO database (GSE13948) using chemically modified antisense oligonucleotides to specifically inhibit endogenous miR-122 in the liver was analyzed, and top 250 DEGs were selected according to GEO2R calculation. Statistically enriched signaling pathways of clustered DEGs were ranked and categorized using DAVID. Gene interaction network of DEGs from selected enriched signaling pathways was achieved using STRING and visualized by Cytoscape 3.0.0 software (www.cytoscape.org) accessed on 25 April 2021.

### 4.10. Data Analyses

All animal experimental data were reported as the mean ± SEM. of the experimental replicates. The differences between groups were analyzed using one-way ANOVA. The statistical significance threshold for all analyses was set at *p* < 0.05 or *p* < 0.01.

## Data Availability

Not applicable.

## Supplementary Materials

The following are available online at https://www.mdpi.com/article/10.3390/ijms22094901/s1.

## Abbreviations

ALT	Alanine aminotransferase
APAP	Acetaminophen
Bax	Bcl-2-associated X protein
DEG	Differentially expressed gene
ER	Endoplasmic reticulum
GEO	Gene expression Omnibus
miR	MicroRNA
mRNA	Messenger RNA
qRT-PCR	Quantitative reverse transcription polymerase chain reaction
TUNEL	Terminal deoxynucleotidyl transferase dUTP nick end labeling
