# Supplementary material for: Hypergravity Load Modulates Acetaminophen Nephrotoxicity via Endoplasmic Reticulum Stress in Association with Hepatic microRNA-122 Expression
Source: Int J Mol Sci. 2021 May 5;22(9):4901. doi: 10.3390/ijms22094901 (PMC8124210; doi:10.3390/ijms22094901)
Supplement: Supplementary file 1 [file ijms-22-04901-s001.zip › ijms-1217086-supplementary.pdf]

Supporting Information

Hypergravity load modulates acetaminophen nephrotoxicity via endoplasmic reticulum stress in association with hepatic microRNA-122 expression

Supporting Results

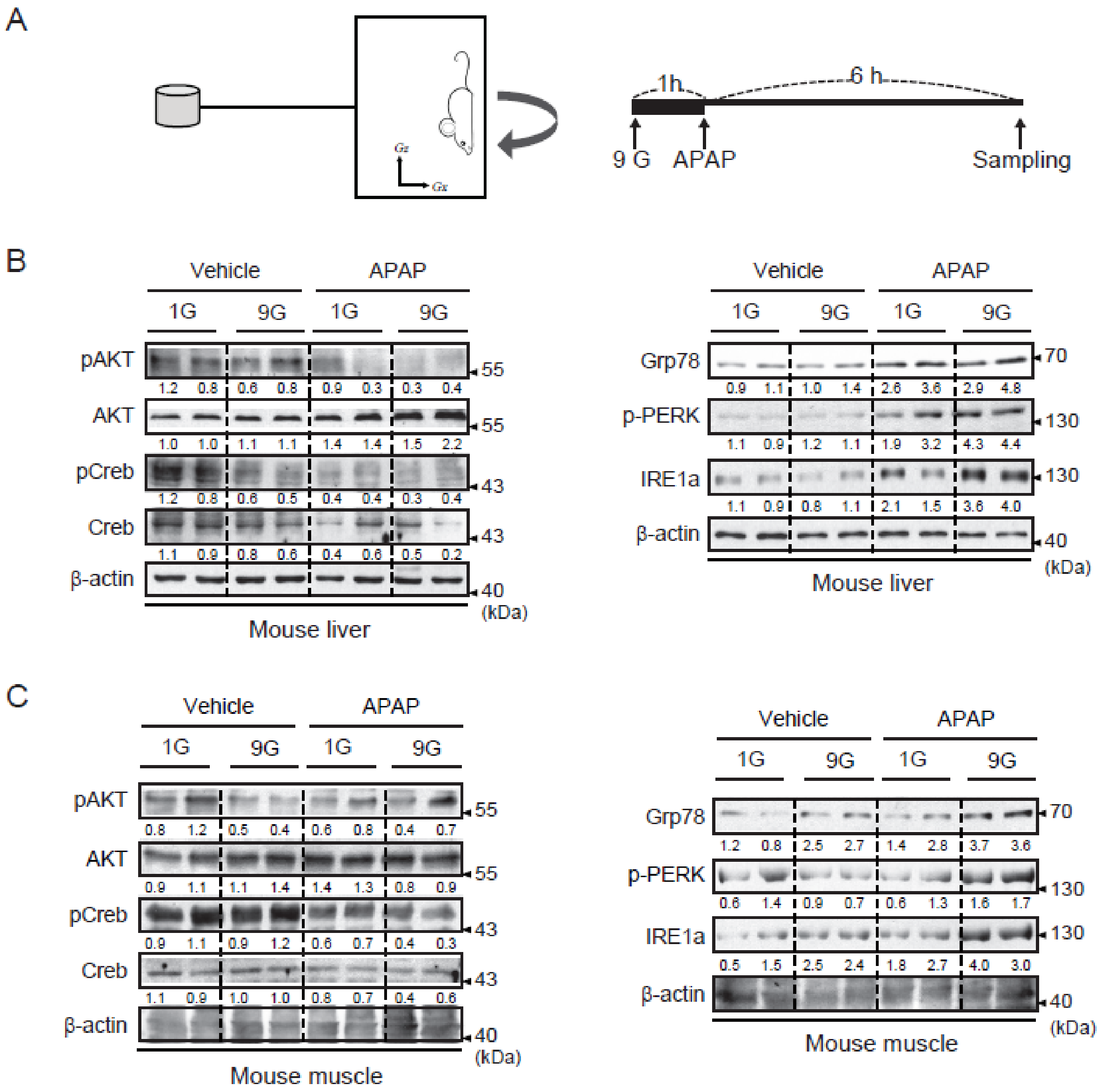

Supplementary Figure 1. Effects of combined treatment with hypergravity and APAP on cell viability and ER stress markers

(A) Schematic of hypergravity and APAP treatments. (B, C) Immunoblots for the pAKT, pCreb, and ER stress markers in the liver and tibialis anterior muscle homogenates. Male C57BL/6 mice were exposed to a single +9 Gx load for 1 h, followed by APAP treatment (500 mg/kg BW, i.p.), then

sacrificed 6 h afterward. Immunoblots were assessed from two samples per condition (randomly selected from 6 samples). The values represent fold changes relative to the control (β-actin).

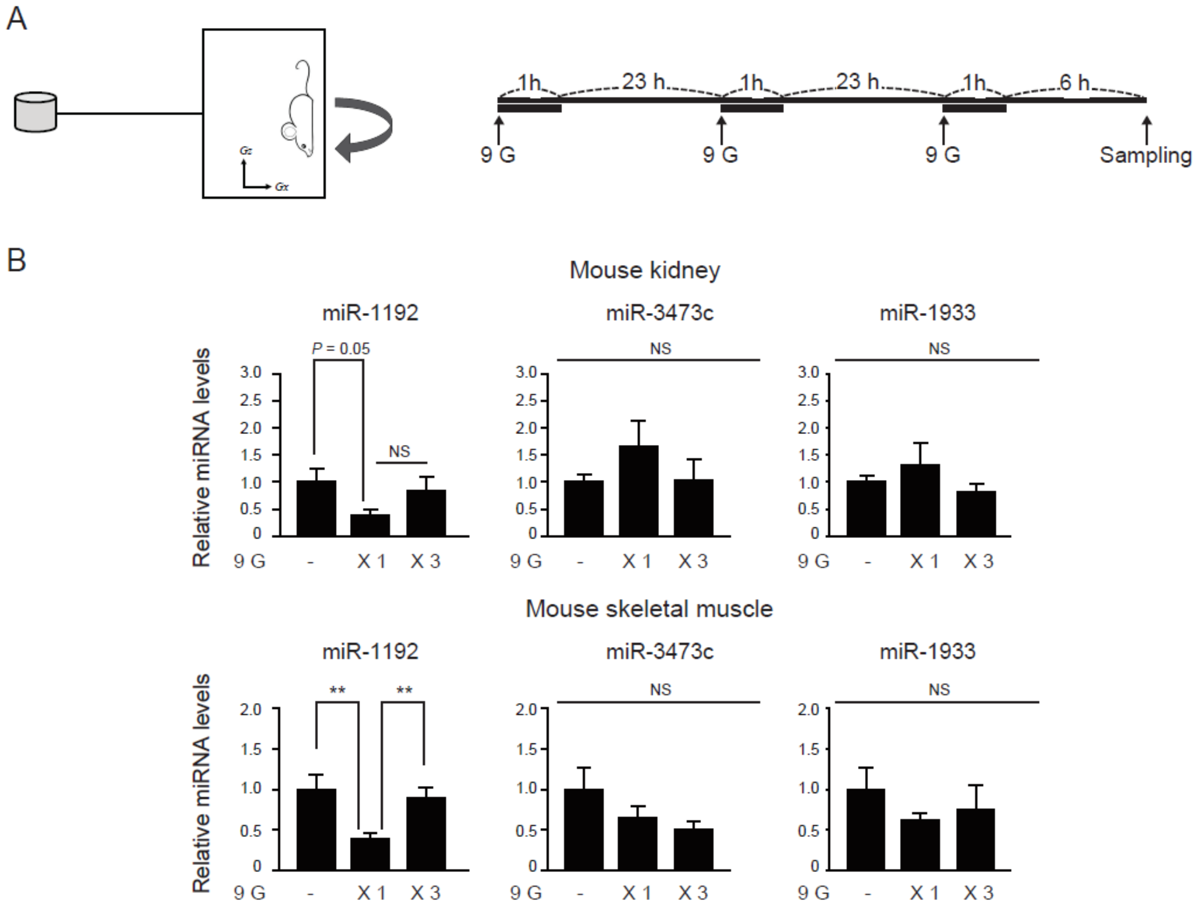

Supplementary Figure 2. Representative miRNA levels in kidney and muscle of mice exposed to a single or multiple hypergravity loads.

(A) Schematic of the single or multiple hypergravity load experiments. (B) qRT-PCR assays for miR-1192, miR-3473c, and miR-1933 in the renal cortex and tibialis anterior muscle of mice subjected to a single or multiple +9 Gx hypergravity loads for 1 h per day (n=6). The data represent the mean ± SEM (statistical differences were determined via the the one-way ANOVA; *P<0.05, **P<0.01, n.s., not significant).

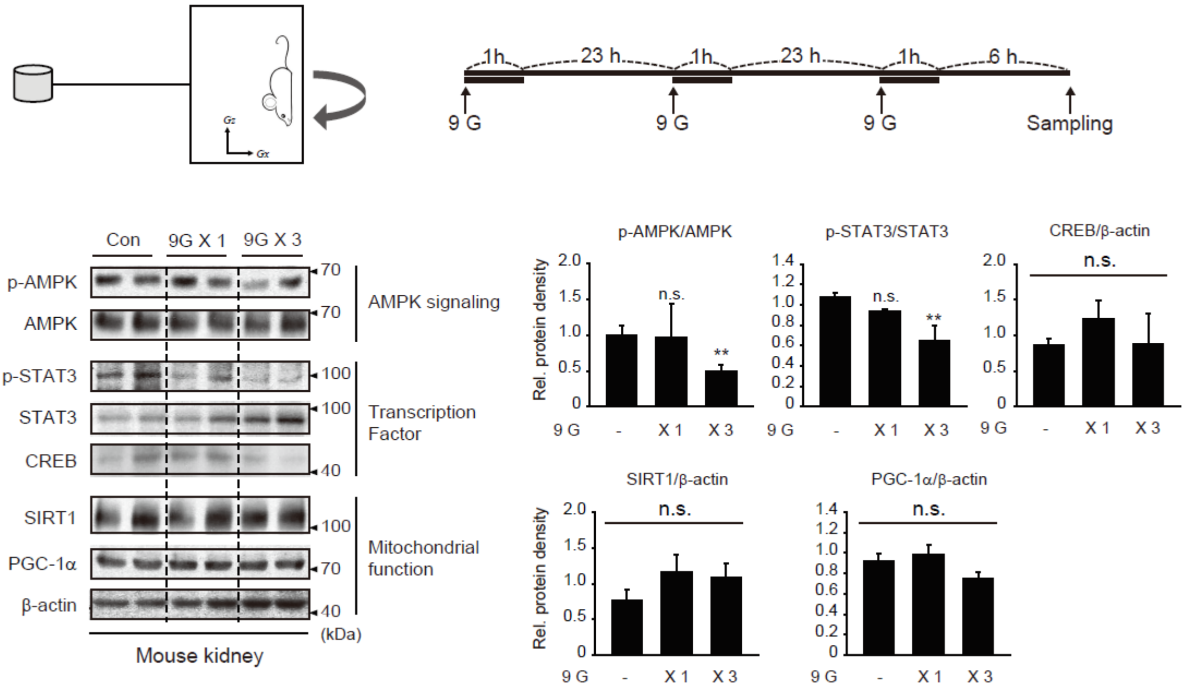

Supplementary Figure 3. Immunoblottings for p-AMPK, p-STAT3, CREB, Sirt1 and PGC-1α in kidney of mice exposed to a single or multiple hypergravity loads.

The samples were obtained using the same mice as in Supplementary Figure 2. Immunoblots were assessed from two samples per condition (randomly selected from 6 samples). The values represent fold changes relative to the control (β-actin).

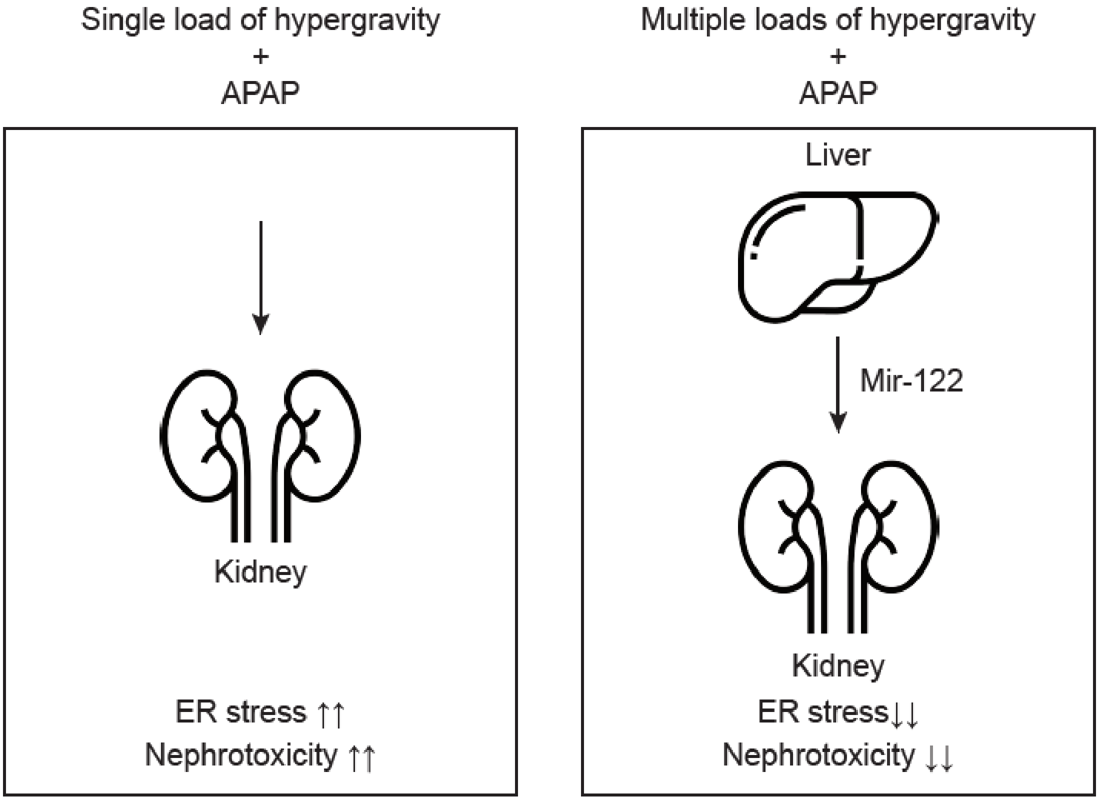

Supplementary Figure 4. Schematic showing the mechanistic basis of kidney protection by multiple hypergravity loads against APAP treatment.

Supporting Table 1. Information on antibodies or other reagents used

Item	Supplier	Catalog no.
pCREB	Cell Signaling	9198
CREB	Cusabio	CSB-PA005947HA01HU
pAKT	Cell Signaling	9275
AKT	Cell Signaling	4685S
Grp78	Abcam	ab21685
p-PERK	Santa Cruz	sc-32577
IRE1a	Santa Cruz	sc-20790
p-AMPK	Cell Signaling	2535S
AMPK	Cell Signaling	2532
β-actin	Sigma	A5441

Supporting Table 2. The sequences of primers

<table>
<tr><th></th><th>Gene symbols</th><th>Forward primers</th><th>Reverse primers</th></tr>
<tr><td rowspan="4">Mouse mRNAs</td><td>Kim-1</td><td>TTCTCCCAGGCACTGTGGAT</td><td>CAGGAATCTCCACTCGACAA</td></tr>
<tr><td>Bax</td><td>AGACAGGGGCCTTTTTGCTAC</td><td>AATTCGCCGGAGACACTCG</td></tr>
<tr><td>Bcl2</td><td>GAGAGCGTCAACAGGGAGATG</td><td>CCAGCCTCCGTTATCCTGGA</td></tr>
<tr><td>Actin</td><td>CTGAGAGGGAAATCGTGC</td><td>TGTTGGCATAGAGGTCTT</td></tr>
<tr><td rowspan="4">microRNAs</td><td>miR-122</td><td>TGGAGTGTGACAATGGTGTTTG</td><td rowspan="4">Universal reverse primer
(Qiagen proprietary information)</td></tr>
<tr><td>miR-1192</td><td>AAACAAACAAACAGACCAAATT</td></tr>
<tr><td>miR-3473c</td><td>TCTCTCCAGCCCCCATAATAAG</td></tr>
<tr><td>miR-1933</td><td>AGTCATGGTGTTCGGTCTTAGTTT</td></tr>
</table>
